# General Nursing Council for Scotland

**Published:** 1921-03-26

**Authors:** 


### General Nursing Council for Scotland.

The meeting of the General Nursing Council for Scotland, held at 13 Melville Street, Edinburgh, on March 16, 1921, had before it documents from the Scottish Board of Health on the subject of the supply of hospital nurses, and the report of a Committee of the Consultative Council on Medical and Allied Services thereon. These documents were remitted to the Education and Examination Committee of the Council for consideration.

A letter was submitted from the Scottish Board of Health with regard to a request received by the Board from certain hospitals that the Board's examination should be continued in order to ensure that nurses commencing training after January 1, 1920, might have their knowledge of anatomy, physiology, and hygiene tested by examination at an early period of their training. The Council were of opinion that the point raised is of importance to all nurses commencing training any considerable time before the Council's examinations are in operation, and that it is not a matter affecting specially the nurses covered hitherto by the Board's examination. After discussion the matter was remitted to the Education and Examination Committee.

Fever Nurses and the General Register.

The Register submitted a summary forwarded by the Scottish Board of Health, showing the total number of fever nurses on the Board's Register to date to be 1,137, of whom 622 had received further training in the larger general infirmaries and hospitals, military hospitals, etc., leaving only 515 nurses on the Board's Fever Register who have fever training only. The Board also pointed out that of these, ninety-three were known to be dead, married, abroad, or not practising. Of the remaining 422 nurses, over 200 obtained the Board's fever certificate as recently as the years 1919 and 1920, and the Board added that it was probable that a considerable number of these were at present undergoing further training in general hospitals, and that the number stated above as dead, married, abroad, etc., is probably far from complete. The Registrar was instructed to thank the Board for the Memorandum, and to state that the Council were pleased to note that so small a number of nurses on the Board's Fever Register would be eligible to apply for admission to the General Register.

It was reported that the Syllabus Committee had adjusted the Questionnaire to be sent to the various hospitals, and that copies were being sent out as soon as possible. It was further reported that this Committee had not yet been able to complete the Draft Syllabus of Training for future nurses, but that it would be completed before the next meeting of the Council.

